# Cerebral Oximetry-Monitored Nitroglycerin Infusion and Tissue Perfusion during Rewarming of Cardiopulmonary Bypass in Cardiac Surgery: A Prospective Randomized Trial

**DOI:** 10.3390/jcm11030712

**Published:** 2022-01-28

**Authors:** Jia-Lin Chen, Yung-Chi Hsu, Go-Shine Huang, Chih-Yuan Lin, Hung-Yen Ke, Po-Shun Hsu, Chi-Hsiang Chung, Chien-Sung Tsai, Tso-Chou Lin

**Affiliations:** 1 Department of Anesthesiology, Tri-Service General Hospital, National Defense Medical Center, Taipei 11490, Taiwan; babe.ane@gmail.com (J.-L.C.); x0939778570@gmail.com (Y.-C.H.); kshgodoc@gmail.com (G.-S.H.); 2 Division of Cardiovascular Surgery, Department of Surgery, Tri-Service General Hospital, National Defense Medical Center, Taipei 11490, Taiwan; linrock@ms26.hinet.net (C.-Y.L.); drkehy@yahoo.com.tw (H.-Y.K.); hsuposhun@gmail.com (P.-S.H.); sung1500@mail.ndmctsgh.edu.tw (C.-S.T.); 3 Department of Medical Research, Tri-Service General Hospital, School of Public Health, National Defense Medical Center, Taipei 11490, Taiwan; g694810042@gmail.com; 4 Department and Graduate Institute of Pharmacology, National Defense Medical Center, Taipei 11490, Taiwan

**Keywords:** cerebral oximetry, nitroglycerin, cardiopulmonary bypass, rewarming, lactate

## Abstract

Background: Nitroglycerin facilitates microcirculation and oxygen delivery through vasodilation. The purpose of this study was to clarify the effects of nitroglycerin-induced vasodilation and potential hypotension on tissue perfusion under cerebral oximetry monitoring during rewarming in cardiopulmonary bypass. Methods: Elective cardiac surgical patients were randomly assigned to either a nitroglycerin group (*n* = 32) with an intravenous infusion of 1–5 mcg/kg/min or a control group (*n* = 31) with 0–0.1 mcg/kg/min infusion, since the initiation of rewarming. Perioperative arterial blood gas data were collected in addition to hemodynamic variables, cerebral oximetry values, urine output, and postoperative outcomes. Results: Nearly one-fifth (6/32) of patients in the nitroglycerin group experienced transient (≤5 min) profound hypotension (mean arterial blood pressure ≤40 mmHg) after the initiation of infusion. There were no significant differences between groups in terms of perioperative levels of cerebral oximetry, cardiac index, plasma glucose, lactate, bicarbonate, base excess, or post-bypass activated coagulation time. In the nitroglycerin group, urine output was nonsignificantly higher during cardiopulmonary bypass (*p* = 0.099) and within 8 h after surgery (*p* = 0.157). Perioperative transfused blood products, postoperative inotropic doses, extubation time, and intensive care unit stay were comparable for the two groups. Conclusions: Initiation of intravenous nitroglycerin infusion (at 1–5 mcg/kg/min) during rewarming in hypothermic cardiopulmonary bypass resulted in transient profound hypotension in one-fifth of patients and did not improve perioperative cerebral oxygenation, tissue perfusion, and coagulation in cardiac surgery.

## 1. Introduction

Cardiopulmonary bypass (CPB) exposes the body to a variety of stresses, including contact of peripheral blood with artificial surfaces of the heart–lung machine, hypothermia, and ischemia-reperfusion injury, and is therefore associated with systemic inflammatory response [1]. Accordingly, endothelial cell dysfunction precipitates decreased production of endogenous nitric oxide, significantly compromising both vascular tone and local tissue perfusion [2]. Low cardiac output and tissue hypoperfusion increase perioperative lactate production before and during hypothermic CPB; consequently, hyperlactatemia and lactic acidosis are commonly encountered during and after cardiac surgery [3,4]. Multivariate analysis has revealed that elevated lactate levels are an independent predictor for postoperative morbidity and mortality in cardiac surgical patients [5].

Nitroglycerin, a nitric oxide donor for vasodilation, has remarkable beneficial effects on microcirculation in patients with heart failure [6] and septic shock [7]. Intravenous low-dose (0.05–0.1 mcg/kg/min) administration of nitroglycerin before and during CPB could prevent a decrease of cerebral oximetry value through vasodilation during CPB [2], but had no significant effect on arterial blood oxygen tension during cardiac surgery [8]. A retrospective study demonstrated that high-dose loading (10–20 mg/h, approximately 2.5–5.0 mcg/kg/min) of nitroglycerin during rewarming attenuated hyperglycemic response and hyperlactatemia during cardiac surgery [9]. However, nitroglycerin-induced vasodilation and potential hypotension raise concerns regarding the lower limits of cerebral blood flow autoregulation. The duration and magnitude of blood pressure below the autoregulation threshold were independently associated with major morbidity or operative mortality after cardiac surgery [10]. Therefore, regional cerebral oximetry monitoring has been recommended during cardiothoracic surgery [11] and applied to guide nitroglycerin infusion during controlled hypotension for major open urological or abdominal surgery [12].

In this study, we prospectively compared the effects of nitroglycerin infusion during rewarming in CPB on perioperative hemodynamic variables, cerebral oximetry values, lactate, and glucose levels for tissue perfusion, coagulation time for vasodilation-mediated heparin reversal, and postoperative short-term outcomes in cardiac surgical patients.

## 2. Materials and Methods

### 2.1. Participants

This study was registered with ClinicalTrials.gov (NCT01901419) after receiving approval from the Institutional Review Board of Tri-Service General Hospital (TSGHIRB-1-102-05-049). Adult patients receiving elective cardiac surgery with hypothermic CPB were recruited from July 2013 to June 2017. Patients were excluded if they had complicating comorbidities, such as chronic hepatic or renal insufficiency, acute cardiopulmonary failure requiring mechanical ventilation, intra-aortic balloon pump, or extracorporeal membrane oxygenation. Patients receiving normothermic CPB according to the surgeon’s decision during operation were also excluded. Before the commencement of this study, statistical power analyses performed with G*Power [13] revealed that a sample size of 27 patients per group was required to achieve 80% power for an effect size of 0.78 in the primary endpoint of post-CPB blood glucose levels [14] because we did not find any previously published data on lactate levels following nitroglycerin infusion during rewarming of CPB. A total of 80 patients were enrolled to allow for patient dropout. Written informed consent was obtained preoperatively.

After initiation of hypothermic CPB, a computer randomization program and sequentially numbered, opaque sealed envelopes were used to assign patients to groups to receive nitroglycerin infusion at either 1–5 mcg/kg/min (nitroglycerin group) or 0–0.1 mcg/kg/min (control group) during later rewarming from CPB. According to Taiwan’s prescribing information for nitroglycerin infusion, a dose of 1–5 mcg/kg/min is recommended for maintaining intraoperative hypotension, whereas 0.05–0.1 mcg/kg/min is appropriate for acute congestive heart failure.

### 2.2. Anesthetic Procedure and Cardiopulmonary Bypass

Routine physiological monitoring, including three-lead electrocardiography, pulse oximetry, and invasive arterial blood pressure monitoring, was performed immediately before anesthesia induction. In addition, cardiac output (FloTrac; Edwards Lifesciences, Irvine, CA, USA) was continuously monitored, and cerebral oximetry based on near-infrared spectroscopy technology (ForeSight cerebral oximeter; CASMED, Branford, CT, USA) was applied on the forehead with a resting baseline value and until the end of surgery.

All patients underwent general anesthesia induced with midazolam (5 mg), fentanyl (1.5–3.0 mcg/kg), propofol (0.5–1.5 mg/kg), and cisatracurium (0.1–0.2 mg/kg), and received sevoflurane or isoflurane for maintenance after tracheal intubation. A pulmonary artery catheter (Swan-Ganz Thermodilution catheter 7.5 Fr; Edwards Lifesciences, CA, USA) was placed through the right internal jugular vein for postoperative monitoring. Real-time cardiac performance was monitored with transesophageal echocardiography throughout the entire procedure. Minute ventilation was adjusted to maintain end-tidal CO_2_ at 40 ± 5 mmHg, confirmed by serial arterial blood gas analyses. After anticoagulation (activated clotting time >350 s) was achieved through intravenous administration of heparin at 300 U/kg prior to cannulation, standard hypothermic CPB (Sarns 8000, Terumo, Ann Arbor, MI, USA) with an extracorporeal membrane oxygenator (Capiox^®^SX 18, Terumo, Ann Arbor, MI, USA) was conducted in sequence to maintain a body temperature of 26 °C–30 °C during surgery. The perfusionist adjusted the pump flow to obtain an adequate output of 2.2 L/m^2^ of body surface area and adjusted the sevoflurane concentration on the vaporizer to maintain a mean arterial blood pressure of 50–80 mmHg.

### 2.3. Treatment Protocol

Before rewarming, patients were randomly assigned to either a nitroglycerin or control group. Nitroglycerin infusion was administered by an unblinded anesthesiologist from the commencement of CPB rewarming until the end of surgery. The infusion rate was tapered off or ceased in response to profound hypotension (mean arterial blood pressure ≤40 mmHg) or cerebral desaturation (absolute saturation value of <50% or relative drop of >20% of baseline value for each side). Following standard rewarming and deaerating, the pump flow was reduced and weaned with routine inotropic support, including dopamine at 3–8 mcg/kg/min and additional dobutamine or epinephrine infusion for acceptable cardiac output. An intra-aortic balloon pump or extracorporeal membrane oxygenation was further applied as required for mechanical circulatory support. Effects of heparin were neutralized with protamine sulfate after bypass cessation. Regular insulin (10 units) was intravenously administered if blood glucose levels exceeded 200 mg/dL.

All patients were transferred to the cardiovascular surgical intensive care unit (ICU) with tracheal intubation after operation. According to routine criteria, extubation was performed after the patient regained oriented consciousness, normothermia (patient rewarmed and shivering controlled), and hemodynamic stability with no uncontrolled arrhythmias and no excessive bleeding (as defined by blood loss <100 mL/h).

### 2.4. Data Acquisition

Blood gas analyses were routinely examined perioperatively with a GEM Premier 3000 (Instrumentation Laboratory, Lexington, MA, USA), including before incision, after heparin administration, before and after aortic unclamping, after protamine administration, and immediately upon ICU admission. Plasma lactate (detectable limit: 0.3–20 mmol/L), bicarbonate, base excess, glucose level (detectable limit: 15–500 mg/dl), and P/F ratio (partial pressure of arterial blood oxygenation/fraction of inspiratory oxygen concentration; PaO_2_/FiO_2_) were analyzed. The ratios of patients with a lactate level of 4.0 mmol/L or higher were calculated [15]. Activated clotting (or coagulation) time was tested by ACT Plus (Medtronic, Minneapolis, MN, USA) before incision, after heparin administration, during CPB, after protamine administration, and before the end of surgery (if needed). Data of perioperative variables, such as arterial blood pressure, cardiac index, urine output, and cerebral oximetry values, were collected. Postoperative outcomes, including use of inotropic agents, time to extubation, lengths of ICU and hospital stay, and 30-day mortality, were also recorded.

### 2.5. Data Analysis

All values are expressed as the mean ± standard deviation. Statistical analysis was conducted using an “intention-to-treat” approach. Mann–Whitney U test was applied to compare means of continuous variables, and Pearson’s chi-square test or Fisher’s exact test was used for categorical variables. Differences with *p* < 0.05 were considered statistically significant. All data analyses were performed using SPSS version 22 (IBM, Armonk, NY, USA).

## 3. Results

### 3.1. Participant Recruitment

**Figure 1:**
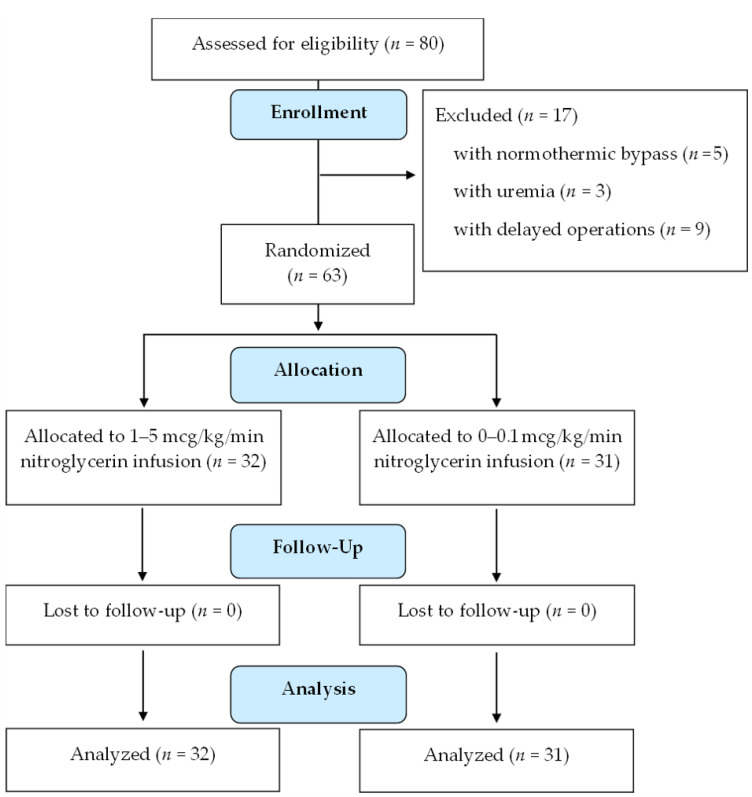
CONSORT flow diagram.

In total (Figure 1), 32 cardiac surgical patients received nitroglycerin infusion at 1–5 mcg/kg/min (nitroglycerin group), and 31 patients received nitroglycerin infusion at 0–0.1 mcg/kg/min (control group).

### 3.2. General Characteristics of Participants

Demographic data are summarized in Table 1. Of the 32 patients in the nitroglycerin group, 6 (18.8%) experienced transient hypotension (mean arterial blood pressure ≤40 mmHg) after the initiation of infusion, and their mean arterial blood pressure recovered to 50 mmHg within 5 min following the immediate tapering of nitroglycerin infusion. The infused nitroglycerin was 3.5 ± 1.9 mg before bypass cessation and 9.8 ± 8.3 mg at the end of surgery. The rewarming rates were comparable in the two groups, with a maximum of 0.44 °C/min in one patient. No mechanical circulatory support was needed after bypass cessation.

### 3.3. Perioperative Outcomes

As shown in Table 2, no significant differences were observed between groups in terms of perioperative plasma lactate, bicarbonate, base excess, or glucose levels. The incidence of perioperative hyperlactatemia (≥4.0 mmol/L) were comparable (all *p* > 0.05). Regular insulin was intravenously administered in three patients in the nitroglycerin group (each 20, 20, and 20 units) and six in the control group (10, 10, 20, 20, 20, and 30 units). In the nitroglycerin group, a nonsignificantly higher amount of urine output was observed during CPB (5.9 ± 3.4 vs. 4.6 ± 3.0 mL/kg/h, *p* = 0.099) and within 8 h after surgery (6.3 ± 3.8 vs. 5.0 ± 2.4 mL/kg/h, *p* = 0.157). Perioperative activated clotting time, transfused blood products, hematocrit, postoperative inotropic doses, extubation time, ICU stay, and hospital stay did not differ significantly between groups. One patient in the nitroglycerin group underwent resternotomy to assess bleeding on the same day, and another one expired on the 25th postoperative day as a result of mediastinitis with septic shock. One patient in the control group developed postoperative pneumonia without further sequelae.

### 3.4. Perioperative Parameters

Table 3 reveals that cardiac index, arterial oxygenation (P/F ratio), and saturation values of cerebral oximetry were comparable in the two groups. Saturation values decreased slightly but nonsignificantly on both the left and right sides following rewarming until bypass cessation compared with the saturation values before rewarming.

## 4. Discussion

### 4.1. Main Findings

Nitroglycerin, a vasodilation agent, facilitates microcirculation and the rewarming process of hypothermic CPB. In this study, intravenous infusion of nitroglycerin at 1–5 mcg/kg/min during rewarming of CPB resulted in transient profound hypotension in nearly one-fifth of patients. Perioperative cerebral oximetry value, blood lactate level, glucose level, and activated coagulation time were comparable to those receiving 0–0.1 mcg/kg/min nitroglycerin infusion. We did not observe any significant benefits of nitroglycerin infusion during rewarming on tissue perfusion and coagulation in cardiac surgery.

### 4.2. Personalized Cerebral Oximetry Monitoring during Hypothermic CPB

**Figure 2:**
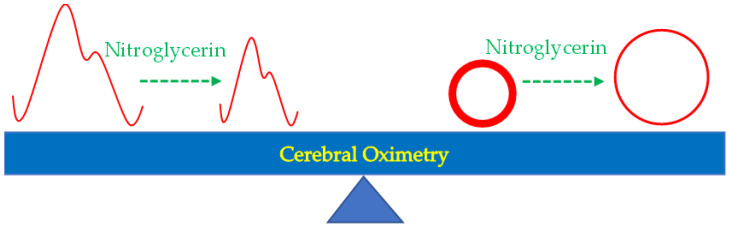
The balance between nitroglycerin-induced hypotension (red traces) and vasodilation (red circles) for tissue perfusion and brain protection under cerebral oximetry monitoring.

Impaired cerebral blood flow autoregulation occurs in 20% of patients during hypothermic CPB [16]. However, there is currently no consensus on the ideal mean arterial pressure and its lower limit during CPB [17]. Notably, arterial atherosclerotic change is ubiquitous in patients with ischemic heart or cerebral diseases. Therefore, real-time autoregulation monitoring using near-infrared spectroscopy may provide a more rational basis for individualizing blood pressure to measure injurious hypotension (mean <45 mmHg) [18] and perioperative stroke [16] during CPB. Precise diagnostic tools can be leveraged to deliver personalized treatments and provide prognostic improvements regarding postoperative morbidity and mortality in cardiac surgery [10,11]. A relative 20% reduction of the baseline value or an absolute value lower than 50% is a suggested threshold for intervention [19,20,21]. In the present study, only one patient in the control group had transient absolute values lower than 50% by the ForeSight^®^ cerebral oximeter during rewarming, and no patients had any neurological sequelae. Therefore, an acceptable cerebral oximetry result following loading of nitroglycerin infusion indicates adequate cerebral tissue oxygenation in the balance between nitroglycerin-induced vasodilation and potential hypotension (Figure 2). An acceptable lower limit of arterial blood pressure during rewarming can thus be individualized according to real-time cerebral autoregulation monitoring.

### 4.3. Hypoperfusion during Hypothermic CPB and Nitroglycerin Infusion for Rewarming

During hypothermic extracorporeal circulation, peripheral vessels are constricted, and tissues are relatively hypoperfused. Inherent cellular hypoxia may increase lactate production. A peak lactate level of 4.0 mmol/L or more during CPB was associated with an increased risk of postoperative morbidity and mortality in cardiac surgery [15]. Therefore, maintenance of mean arterial blood pressure and pump flow to preserve blood flow to vital organs requires either adequate intravenous volume or vascular resistance. Alternatively, anesthetic depth may be adjusted lower to trigger the release of catecholamines for vascular tone. Among patients with inadequate anesthetic status during the CPB period, rapid nitroglycerin infusion usually results in immediate hypotension following vasodilation. Nitroglycerin infusion at a rate of 1–5 mcg/kg/min, recommended for maintenance of intraoperative hypotension, has been clinically used to manipulate blood pressure in cardiac surgery, especially prior to aortic cannulation of CPB. In our study, initiation of nitroglycerin infusion resulted in a transient decline in mean arterial blood pressure to less than 40 mmHg in nearly one-fifth of our patients with spontaneous recovery to 50 mmHg within 5 min after tapering off nitroglycerin infusion. Tai et al. [9] retrospectively analyzed the metabolic effects of intravenous loading of nitroglycerin (infusion rate: 10–20 mg/h, with total dose: 0.5 mg/kg) during rewarming and after CPB. The result was that it effectively attenuated hyperglycemic response and reduced the incidence of hyperlactatemia in cardiac surgery, but they did not record perioperative hypotensive events. In the present study, the total dose administered to patients in the nitroglycerin group was 0.05 mg/kg before CPB cessation and 0.15 mg/kg before the end of surgery, which was much lower than in the aforementioned study, and would explain the comparable clinical parameters between the high-dose infusion and control groups. A prospective study demonstrated that nitroglycerin infusion at 0.5 mcg/kg/min during rewarming improved neither peripheral-core temperature gradient after CPB nor postoperative lactate concentrations [22], consistent with our results. Volume status and anesthetic depth during rewarming, as well as concerns among cardiac surgeons and perfusionists of hypotension, may interfere with the use of high-dose nitroglycerin infusion, which should be considered when elaborating the technique’s actual clinical benefits.

### 4.4. Heparin Reversal and Nitroglycerin Infusion

During cardiac surgery, intravenous heparin binds to antithrombin for anticoagulation and can be monitored according to the activated clotting time and eventually reversed by protamine [23]. During hypothermic CPB, heparin bound to various plasma proteins and endothelial cells may become trapped in constricted peripheral vessels and might not be sufficiently metabolized and eliminated following rewarming. Consequently, inadequate reversal by protamine after bypass cessation tends to cause postoperative bleeding. Nitroglycerin infusion enhances peripheral vasodilation to theoretically facilitate rewarming and release of trapped heparin. Early detection and correction of anticoagulant status would ameliorate intraoperative and postoperative bleeding. However, we did not observe significant differences in activated clotting time or transfused blood products following CPB in our patients.

### 4.5. Limitations

Three limitations to our study should be addressed. First, this preliminary study was conducted in a medical center with a limited number of cases. Nitroglycerin infusion has been routinely used in our clinical practice during cardiac surgery. However, its dose-dependent vasodilatory effects on cerebral oxygenation, heparin reversal, and metabolic homeostasis, such as blood glucose and lactate levels, were scarcely reported in previous studies when we commenced this prospective study. Therefore, the significantly different glucose levels in our previous study [14] were substituted for lactate levels as the primary endpoint of tissue perfusion for the calculation of the sample size. However, based on the present results from more than 30 patients in each group, we observed that most clinical variables were closely comparable. The study was then terminated due to a lack of anticipated significant difference within an acceptable sample size. Second, to maintain a mean arterial blood pressure above 50 mmHg, the maximal infusion rate of nitroglycerin was mostly 1–2 mcg/kg/min, despite the high-dose infusion of 1–5 mcg/kg/min in the nitroglycerin group. In our clinical practice, cardiac surgeons and perfusionists actively manage hemodynamic maintenance during cardiopulmonary bypass. Monitoring cerebral oximetry results and checking for further positive neurological outcomes may reduce the surgeon’s concerns and promote the use of adequate nitroglycerin infusion in both clinical practice and future studies. Third, we monitored blood pressure and laboratory data rather than tissue microcirculation in this study, largely because satisfactory blood pressure is more easily achieved than adequate microcirculation in clinical practice. Cardiac surgical patients under CPB have some hemodynamic characteristics similar to those of septic patients, including systemic inflammatory status, low vascular resistance, and impaired microcirculation. After a 0.5 mg bolus of nitroglycerin, microcirculation in septic patients was reported to improve rapidly in 2 min [24]; however, a 30 min infusion of nitroglycerin at 4 mg/h (close to 1 mcg/kg/min) yielded results comparable with a placebo in a randomized trial of 70 patients with septic shock [25]. At present, nitroglycerin infusion provides only transient vasodilation for better microcirculation, and further studies are required to investigate its sustained effects on global tissue perfusion and homeostasis in cardiac surgery.

## 5. Conclusions

We did not observe a significant effect of intravenous nitroglycerin infusion at 1–5 mcg/kg/min during rewarming of cardiopulmonary bypass on cerebral oxygenation, tissue perfusion, or reversal of heparin-related coagulation in cardiac surgical patients. Cerebral oximetry monitoring provides precise diagnostic brain protection during transient profound hypoperfusion after initiation of nitroglycerin infusion.

## Figures and Tables

**Table 1:** Patient demographic data.

	Nitroglycerin 1–5 mcg/kg/min (*n* = 32)	Nitroglycerin 0–0.1 mcg/kg/min (*n* = 31)	*p*-Value
Male/female, *n*	22/10	21/10	0.999 ^a^
Age, year	62.5 ± 12.2	59.3 ± 13.2	0.389 ^b^
Weight, kg	63.5 ± 11.9	68.4 ± 17.6	0.271 ^b^
Height, cm	162.9 ± 7.9	164.7 ± 9.7	0.413 ^b^
Body mass index, kg/m^2^	23.8 ± 3.4	24.9 ± 4.7	0.375 ^b^
Nitroglycerin infusion, mg			
Before cessation of CPB	3.5 ± 1.9	0.2 ± 0.3	<0.001 ^b^
Total, until the end of surgery	9.8 ± 8.3	2.1 ± 4.0	<0.001 ^b^
Duration of general anesthesia, min	348.9 ± 64.7	347.3 ± 87.9	0.736 ^b^
Duration of operation, min	291.7 ± 63.9	289.6 ± 82.5	0.680 ^b^
Duration of CPB, min	133.3 ± 48.4	123.3 ± 28.4	0.874 ^b^
Duration of aortic clamping, min	86.8 ± 36.0	78.6 ± 22.2	0.523 ^b^
Lowest temperature during CPB, °C	27.2 ± 1.2	27.6 ± 2.3	0.951 ^b^
Temperature at start of rewarming, °C	28.8 ± 1.3	27.6 ± 2.3	0.563 ^b^
Temperature at end of rewarming, °C	36.4 ± 0.8	36.4 ± 0.9	0.700 ^b^
Duration of rewarming, min	30.5 ± 7.9	34.2 ± 8.5	0.057 ^b^
Rate of rewarming, °C/min	0.3 ± 0.1	0.2 ± 0.1	0.314 ^b^
Fentanyl, mcg/kg	6.5 ± 3.2	4.5 ± 2.2	0.007 ^b^
Packed red blood cell, unit	4.1 ± 1.6	5.0 ± 3.5	0.361 ^b^
Single donor platelet, unit	1.2 ± 0.5	1.2 ± 0.5	0.819 ^b^
Fresh frozen plasma, unit	8.1 ± 0.8	8.1 ± 1.7	0.649 ^b^
Left ventricular ejection fraction, %	57.8 ± 16.1	53.3 ± 14.6	0.166 ^b^
Coronary artery graft in bypass surgery, *n*	0.9 ± 1.3	0.9 ± 1.3	0.847 ^b^
Valvular heart disease, *n*	24	22	0.782 ^a^
Hypertension with medication, *n*	19	18	0.916 ^a^
Diabetes mellitus, *n*	8	9	0.782 ^a^
Perioperative use of insulin, *n*	3	6	0.302 ^a^

Data are presented as the mean ± standard deviation or number. CPB, cardiopulmonary bypass. ^a^, *p*-values estimated with Fisher’s exact test; ^b^, *p*-values estimated with Mann–Whitney U test.

**Table 2:** Perioperative variables and postoperative outcomes.

	Nitroglycerin 1–5 mcg/kg/min (*n* = 32)	Nitroglycerin 0–0.1 mcg/kg/min (*n* = 31)	*p*-Value
Plasma lactate level, mmol/L			
Before incision	1.1 ± 0.5	1.0 ± 0.4	0.921 ^b^
Before start of CPB	1.4 ± 0.7	1.6 ± 0.7	0.315 ^b^
Release of aortic clamp	3.1 ± 1.2	2.6 ± 1.0	0.081 ^b^
After protamine administration	3.3 ± 1.2	3.0 ± 1.1	0.496 ^b^
Upon arrival at ICU	3.4 ± 1.6	3.2 ± 1.5	0.492 ^b^
Peak level in ICU	5.0 ± 3.0	4.1 ± 2.2	0.189 ^b^
Time to peak level in ICU, hour	5.1 ± 5.6	5.0 ± 5.8	0.889 ^b^
Plasma lactate level ≥4.0 mmol/L, *n*			
Before incision	0	0	-
Before start of CPB	0	0	-
Release of aortic clamp	7	4	0.509 ^a^
After protamine administration	8	6	0.763 ^a^
Upon arrival at ICU	10	8	0.782 ^a^
Plasma bicarbonate level, mmol/L			
Before incision	25.9 ± 2.8	26.3 ± 2.2	0.842 ^b^
Before start of CPB	23.9 ± 3.3	23.8 ± 2.9	0.864 ^b^
Release of aortic clamp	22.8 ± 1.8	23.2 ± 1.6	0.441 ^b^
After protamine administration	24.0 ± 2.2	24.3 ± 1.9	0.527 ^b^
Upon arrival at ICU	26.3 ± 2.9	25.1 ± 3.4	0.137 ^b^
Plasma base excess level, mmol/L			
Before incision	2.9 ± 2.4	2.4 ± 2.1	0.153 ^b^
Before start of CPB	−1.3 ± 4.4	−0.7 ± 3.7	0.564 ^b^
Release of aortic clamp	−1.4 ± 1.9	−1.2 ± 1.8	0.869 ^b^
After protamine administration	−0.3 ± 1.9	0.4 ± 2.0	0.263 ^b^
Upon arrival of ICU	1.5 ± 3.1	0.2 ± 3.0	0.052 ^b^
Plasma glucose level, mg/dL			
Before incision	139.4 ± 47.7	124.2 ± 32.0	0.248 ^b^
Before start of CPB	179.2 ± 62.3	165.7 ± 46.2	0.583 ^b^
Release of aortic clamp	174.5 ± 49.5	165.0 ± 29.3	0.487 ^b^
After protamine administration	196.9 ± 53.8	205.7 ± 41.1	0.574 ^b^
Upon arrival at ICU	206.4 ± 55.7	214.8 ± 46.3	0.514 ^b^
Hematocrit, %			
Before incision	38.4 ± 5.9	38.9 ± 5.2	0.853 ^b^
Before start of CPB	28.4 ± 8.3	32.4 ± 8.7	0.036 ^b^
Release of aortic clamp	23.2 ± 2.5	23.5 ± 4.0	0.994 ^b^
After protamine administration	27.7 ± 2.6	29.0 ± 3.7	0.067 ^b^
Upon arrival at ICU	30.8 ± 3.9	31.1 ± 4.2	0.918 ^b^
Activated clotting time			
Before incision, sec	158.9 ± 19.8	153.1 ± 18.1	0.350 ^b^
After protamine administration, sec	144.2 ± 15.9	138.2 ± 15.3	0.212 ^b^
Serum creatinine level, mg/dL			
Preoperative	1.0 ± 0.3	1.0 ± 0.6	0.728 ^b^
Postoperative, upon arrival at ICU	0.9 ± 0.2	1.0 ± 0.5	0.728 ^b^
Postoperative, the next day in ICU	1.1 ± 0.4	1.2 ± 0.6	0.934 ^b^
Intraoperative total furosemide, mg/kg	1.1 ± 0.3	1.2 ± 0.6	0.772 ^b^
Urine output during CPB, mL/kg/h	5.9 ± 3.4	4.6 ± 3.0	0.099 ^b^
Urine output within 8 h after surgery, mL/kg/h	6.3 ± 3.8	5.0 ± 2.4	0.157 ^b^
Postoperative dopamine, mcg/kg/min	3.7 ± 1.8	4.7 ± 2.0	0.053 ^b^
Postoperative dobutamine, mcg/kg/min	0.7 ± 1.7	1.4 ± 2.6	0.406 ^b^
Time to extubation since arrival at ICU, hour	43.6 ± 33.7	45.8 ± 34.8	0.816 ^b^
Length of ICU stay, day	4.1 ± 1.8	4.0 ± 2.0	0.665 ^b^
Length of hospital stay, day	15.6 ± 9.0	14.6 ± 7.4	0.745 ^b^
Postoperative events			
Resternotomy for bleeding, *n*	1	0	0.508 ^a^
Mediastinitis with septic shock, *n*	1	0	0.508 ^a^
Pneumonia, *n*	0	1	0.492 ^a^
Massive hemothorax	0	1	0.492 ^a^
Mortality in 30 days, *n*	1	0	0.508 ^a^

Data are presented as the mean ± standard deviation or number. CPB, cardiopulmonary bypass; ICU, intensive care unit. ^a^, *p*-values estimated with Fisher’s exact test; ^b^, *p*-values estimated with Mann–Whitney U test.

**Table 3:** Perioperative cardiac index and cerebral oximetry values.

	Nitroglycerin 1–5 mcg/kg/min (*n* = 32)	Nitroglycerin 0–0.1 mcg/kg/min (*n* = 31)	*p*-Value
Cardiac index, L/min/m^2^			
Before start of CPB	3.0 ± 1.3	2.8 ± 1.1	0.755 ^b^
Cessation of CPB	2.6 ± 1.0	2.7 ± 0.6	0.411 ^b^
After protamine administration	3.2 ± 1.0	3.1 ± 0.7	0.860 ^b^
Upon arrival at ICU	3.3 ± 0.9	3.3 ± 1.3	0.540 ^b^
PaO_2/_FiO_2_ ratio			
Before start of CPB	420.3 ± 75.6	356.1 ± 90.0	0.010 ^b^
Release of aortic clamp	322.0 ± 93.2	317.7 ± 102.4	0.694 ^b^
After protamine administration	309.1 ± 106.0	311.4 ± 126.6	0.929 ^b^
Upon arrival at ICU	220.1 ± 126.0	194.5 ± 92.4	0.406 ^b^
Cerebral oximetry			
Before start of CPB			
Left	74.4 ± 4.8	76.8 ± 6.9	0.118 ^b^
Right	74.9 ± 4.6	77.9 ± 6.0	0.025 ^b^
Beginning rewarming			
Left	68.0 ± 5.7	68.4 ± 7.8	0.968 ^b^
Right	67.9 ± 7.2	69.5 ± 8.3	0.476 ^b^
Rewarming to 32 °C			
Left	67.1 ± 5.6	65.6 ± 6.8	0.315 ^b^
Right	67.2 ± 6.7	66.9 ± 7.9	0.813 ^b^
5 min after rewarming to 32 °C			
Left	67.4 ± 5.1	65.5 ± 6.9	0.260 ^b^
Right	67.9 ± 5.8	66.9 ± 7.5	0.457 ^b^
Release of aortic clamp			
Left	67.4 ± 5.6	66.8 ± 5.8	0.423 ^b^
Right	68.2 ± 6.1	67.9 ± 6.7	0.804 ^b^
Cessation of CPB			
Left	71.1 ± 4.0	69.8 ± 5.8	0.451 ^b^
Right	71.4 ± 4.9	70.0 ± 6.6	0.471 ^b^
After protamine administration			
Left	74.9 ± 4.3	72.1 ± 6.9	0.173 ^b^
Right	75.0 ± 4.7	74.3 ± 7.3	0.823 ^b^

Data are presented as the mean ± standard deviation. CPB, cardiopulmonary bypass; ICU, intensive care unit; PaO_2_/FiO_2_, partial pressure of arterial blood oxygenation/fraction of inspiratory oxygen concentration. ^b^, *p*-values estimated with Mann–Whitney U test.

## Data Availability

The data presented in this study are available on request from the corresponding author.

## References

[1] Laffey J.G., Boylan J.F., Cheng D.C. (2002). The systemic inflammatory response to cardiac surgery: Implications for the anesthesiologist. Anesthesiology.

[2] Piquette D., Deschamps A., Belisle S., Pellerin M., Levesque S., Tardif J.C., Denault A.Y. (2007). Effect of intravenous nitroglycerin on cerebral saturation in high-risk cardiac surgery. Can. J. Anaesth..

[3] O’Connor E., Fraser J.F. (2012). The interpretation of perioperative lactate abnormalities in patients undergoing cardiac surgery. Anaesth. Intensive Care.

[4] Hsu Y.C., Hsu C.H., Huang G.S., Lu C.C., Wu Z.F., Tsai Y.T., Lin C.Y., Lin Y.C., Tsai C.S., Lin T.C. (2015). Extreme hyperlactatemia after heart transplantation: One center’s experience. Transpl. Proc..

[5] Hajjar L.A., Almeida J.P., Fukushima J.T., Rhodes A., Vincent J.L., Osawa E.A., Galas F.R. (2013). High lactate levels are predictors of major complications after cardiac surgery. J. Thorac. Cardiovasc. Surg..

[6] Lima A., van Genderen M.E., van Bommel J., Klijn E., Jansem T., Bakker J. (2014). Nitroglycerin reverts clinical manifestations of poor peripheral perfusion in patients with circulatory shock. Crit. Care.

[7] den Uil C.A., Caliskan K., Lagrand W.K., van der Ent M., Jewbali L.S., van Kuijk J.P., Spronk P.E., Simoons M.L. (2009). Dose-dependent benefit of nitroglycerin on microcirculation of patients with severe heart failure. Intensive Care Med..

[8] Masoumi G., Pour E.H., Sadeghpour A., Ziayeefard M., Alavi M., Anbardan S.J., Shirani S. (2012). Effect of different dosages of nitroglycerin infusion on arterial blood gas tensions in patients undergoing on- pump coronary artery bypass graft surgery. J. Res. Med. Sci..

[9] Tai Y.H., Chang K.Y., Liao S.W., Chung K.C., Shih C.C., Ho S.T., Lu C.C., Tsou M.Y. (2016). Intravenous loading of nitroglycerin during rewarming of cardiopulmonary bypass improves metabolic homeostasis in cardiac surgery: A retrospective analysis. J. Anesth..

[10] Ono M., Brady K., Easley R.B., Brown C., Kraut M., Gottesman R.F., Hogue C.W. (2014). Duration and magnitude of blood pressure below cerebral autoregulation threshold during cardiopulmonary bypass is associated with major morbidity and operative mortality. J. Thorac. Cardiovasc. Surg..

[11] Juliana N., Abu Yazit N.A., Kadiman S., Muhammad Hafidz K., Azmani S., Mohd Fahmi Teng N.I., Das S. (2021). Intraoperative cerebral oximetry in open heart surgeries reduced postoperative complications: A retrospective study. PLoS ONE.

[12] Van Noord B.A., Stalker C.L., Roffey P., Thangathurai D. (2014). The use of regional cerebral oximetry monitoring during controlled hypotension: A case series. J. Clin. Monit. Comput..

[13] Faul F., Erdfelder E., Lang A.G., Buchner A. (2007). G*Power 3: A flexible statistical power analysis program for the social, behavioral, and biomedical sciences. Behav. Res. Methods.

[14] Hsu C.H., Hsu Y.C., Huang G.S., Lu C.C., Ho S.T., Liaw W.J., Tsai Y.T., Lin C.Y., Tsai C.S., Lin T.C. (2016). Isoflurane compared with fentanyl-midazolam-based anesthesia in patients undergoing heart transplantation: A retrospective cohort study. Medicine.

[15] Demers P., Elkouri S., Martineau R., Couturier A., Cartier R. (2000). Outcome with high blood lactate levels during cardiopulmonary bypass in adult cardiac operation. Ann. Thorac. Surg..

[16] Ono M., Joshi B., Brady K., Easley R.B., Zheng Y., Brown C., Baumgartner W., Hogue C.W. (2012). Risks for impaired cerebral autoregulation during cardiopulmonary bypass and postoperative stroke. Br. J. Anaesth..

[17] Joshi B., Ono M., Brown C., Brady K., Easley R.B., Yenokyan G., Gottesman R.F., Hogue C.W. (2012). Predicting the limits of cerebral autoregulation during cardiopulmonary bypass. Anesth. Analg..

[18] Brady K., Joshi B., Zweifel C., Smielewski P., Czosnyka M., Easley R.B., Hogue C.W. (2010). Real-time continuous monitoring of cerebral blood flow autoregulation using near-infrared spectroscopy in patients undergoing cardiopulmonary bypass. Stroke.

[19] Deschamps A., Lambert J., Couture P., Rochon A., Lebon J.-S., Ayoub C., Cogan J., Denault A. (2013). Reversal of decreases in cerebral saturation in high-risk cardiac surgery. J. Cardiothorac. Vasc. Anesth..

[20] Slater J.P., Guarino T., Stack J., Vinod K., Bustami R.T., Brown J.M., Rodriguez A.L., Magovern C.J., Zaubler T., Freundlich K. (2009). Cerebral oxygen desaturation predicts cognitive decline and longer hospital stay after cardiac surgery. Ann. Thorac. Surg..

[21] Jo Y.Y., Shim J.K., Soh S., Suh S., Kwak Y.L. (2020). Association between cerebral oxygen saturation with outcome in cardiac surgery: Brain as an index organ. J. Clin. Med..

[22] Mullane D., Lenihan M., Hanley C., Wall T., Bukowska I., Griffin M., Flood G. (2020). Efficacy of Glyceryl trinitrate (GTN) to facilitate the rewarming process during cardiopulmonary bypass. J. Cardiothorac. Surg..

[23] Finley A., Greenberg C. (2013). Review article: Heparin sensitivity and resistance: Management during cardiopulmonary bypass. Anesth. Analg..

[24] Spronk P.E., Ince C., Gardien M.J., Mathura K.R., Oudemans-van Straaten H.M., Zandstra D.F. (2002). Nitroglycerin in septic shock after intravascular volume resuscitation. Lancet.

[25] Boerma E.C., Koopmans M., Konijn A., Kaiferova K., Bakker A.J., van Roon E.N., Buter H., Bruins N., Egbers P.H., Gerritsen R.T. (2010). Effects of nitroglycerin on sublingual microcirculatory blood flow in patients with severe sepsis/septic shock after a strict resuscitation protocol: A double-blind randomized placebo controlled trial. Crit. Care Med..

